# Microbiome‐Based Clustering Identifies Glycemic Control‐Related Subtypes in Youth With Recent‐Onset Type 1 Diabetes

**DOI:** 10.1002/mco2.70705

**Published:** 2026-03-28

**Authors:** Huiling Tan, Yu Ding, Zhaohe Gu, Xulin Wang, Jing Wang, Tian Wei, Xiaoya Zhang, Lanxin Pan, Yu Shi, Shiru Chang, Chuang Guo, Jianping Weng, Xueying Zheng, Tong Yue

**Affiliations:** ^1^ Department of Endocrinology and Metabolism The First Affiliated Hospital of USTC, University of Science and Technology of China Hefei Anhui China; ^2^ Department of Rheumatology and Immunology Institute of Endocrine and Metabolic Diseases The First Affiliated Hospital of USTC Division of Life Sciences and Medicine University of Science and Technology of China Hefei Anhui China; ^3^ Department of Endocrinology and Metabolism, Centre for Leading Medicine and Advanced Technologies of IHM The First Affiliated Hospital of USTC, Division of Life Sciences and Medicine, University of Science and Technology of China Hefei Anhui China

**Keywords:** endotype, glycemic control, gut microbiota, machine learning, multi‐omics, type 1 diabetes

## Abstract

Type 1 diabetes (T1D) in children exhibits substantial heterogeneity in glycemic control, yet the biological mechanisms underlying this variation remain unclear. We aimed to explore endotype heterogeneity in youth with recent‐onset T1D using unsupervised clustering based on multi‐omics data, and to identify associated molecular signatures and underlying mechanisms. In a discovery cohort of 69 children and adolescents with recent‐onset T1D, unsupervised clustering of fecal metagenomic profiles revealed two robust subgroups distinguished by hemoglobin A1c (HbA1c) levels. The High‐HbA1c group was enriched in *Bacteroidota*, while the Low‐HbA1c group was enriched in *Firmicutes* and certain *Bacteroides* species (*Bacteroides ovatus*, *Bacteroides xylanisolvens*, *Bacteroides nordii*, and *Bacteroides cellulosilyticus*). Metabolomics revealed significant enrichment of tryptophan‐derived metabolites in the Low‐HbA1c group. *Bacteroides* species signatures are positively correlated with tryptophan metabolite skatole. In an independent validation cohort, *Bacteroides* signatures discriminated individuals with good versus poor glycemic control (AUC = 0.854). Similar microbial patterns were observed in healthy children stratified by glycemic risk, indicating broader relevance of these signatures. Together, microbiome‐based clustering identified glycemic control‐related subtypes in T1D youth and suggested a potential role of *Bacteroides* and skatole in glycemic control. Mechanistic studies are warranted to confirm its role as a glycemic control‐related endotype with distinct pathophysiology.

## Graphical abstract

Microbiome‐based clustering investigated possible glycemic control‐related endotypes in youth with recent‐onset T1D, and found a potential role of *Bacteroides* and skatole production in glycemic control. This study offers novel perspectives on the influence of intestinal microbes and its metabolites in T1D heterogeneity and lays a foundation for future studies investigating microbiome‐targeted strategies for personalized glycemic control.

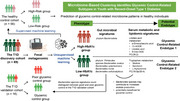

## 1 Introduction

Type 1 diabetes (T1D) is a clinically and biologically heterogeneous autoimmune disease characterized by considerable variability in etiopathogenesis and treatment responses, posing significant challenges to effective disease management [1]. Previous research has established that glycemic control in children with T1D is closely linked to their long‐term risk of diabetes‐related complications [2]. Nevertheless, the majority of pediatric patients under current T1D care fail to achieve the glycemic targets recommended by the International Society for Pediatric and Adolescent Diabetes [3]. This gap is largely attributable to the marked heterogeneity in glycemic control observed across individuals with T1D [4]. Consequently, developing more tailored therapeutic strategies based on individualized glycemic profiles is essential to further improve clinical outcomes in this population.

A promising strategy for driving precision medicine in diabetes is the identification of disease endotypes, which represent mechanistically distinct subtypes of T1D defined by specific pathogenic pathways rather than external clinical manifestations. Unlike clinical phenotypes, which reflect observable disease features, endotypes capture the underlying molecular characteristics and mechanistic pathways that may be amenable to tailored therapeutic interventions [1]. Previous studies have classified T1D patients into Endotype 1 and Endotype 2 based on β‐cell destruction, immune cell infiltration in insulitis, and proinsulin processing, with these endotypes showing distinct clinical phenotypes, particularly in age at disease onset and treatment responses [5]. However, whether more endotypes associated with specific phenotypes and clinical outcomes exist remains to be explored [6]. A critical gap remains in understanding how precisely these endotypes relate to clinical outcomes. The mechanisms underlying their differences are poorly characterized, limiting the translation of these findings into therapeutic strategies.

T1D develops in people with inherited susceptibility under the influence of environmental factors. Among these, the gut microbiome and microbiome‐derived metabolites are now considered key environmental factors to T1D pathogenesis and progression [7]. However, to date, research on T1D endotypes based on gut microbiome features remains scarce. Most existing studies have focused on differences in gut microbial composition across clinical phenotypes, such as levels of glycemic control. Furthermore, these studies have largely examined adult or long‐duration T1D patients [8, 9, 10, 11], with relatively limited attention to recently diagnosed children and adolescents. In addition, many prior investigations have relied on 16S rRNA sequencing, which lacks the taxonomic and functional resolution offered by metagenomic approaches [11]. To date, only one INNODIA study has used metagenomic sequencing to report gut microbiome alterations associated with glycemic control in children and adolescents with recent‐onset T1D [12]. However, this study did not integrate multi‐omics data to further explore underlying molecular mechanisms.

Here, we applied a data‐driven approach to investigate T1D endotype heterogeneity from the perspective of the gut microbiome. Unsupervised clustering of fecal metagenomic data revealed two distinct T1D clusters, which exhibited significant differences in hemoglobin A1c (HbA1c) levels. We further identify gut microbial signatures, serum metabolic signatures, and lipidomic signatures between the groups, and performed multi‐omics correlation analyses to discover potential pathways associated with glycemic control.

## 2 Results

### 2.1 Unsupervised Clustering Identified High‐ and Low‐HbA1c Groups Based on Fecal Metagenomic Profiles in the T1D Discovery Cohort

**FIGURE 1:**
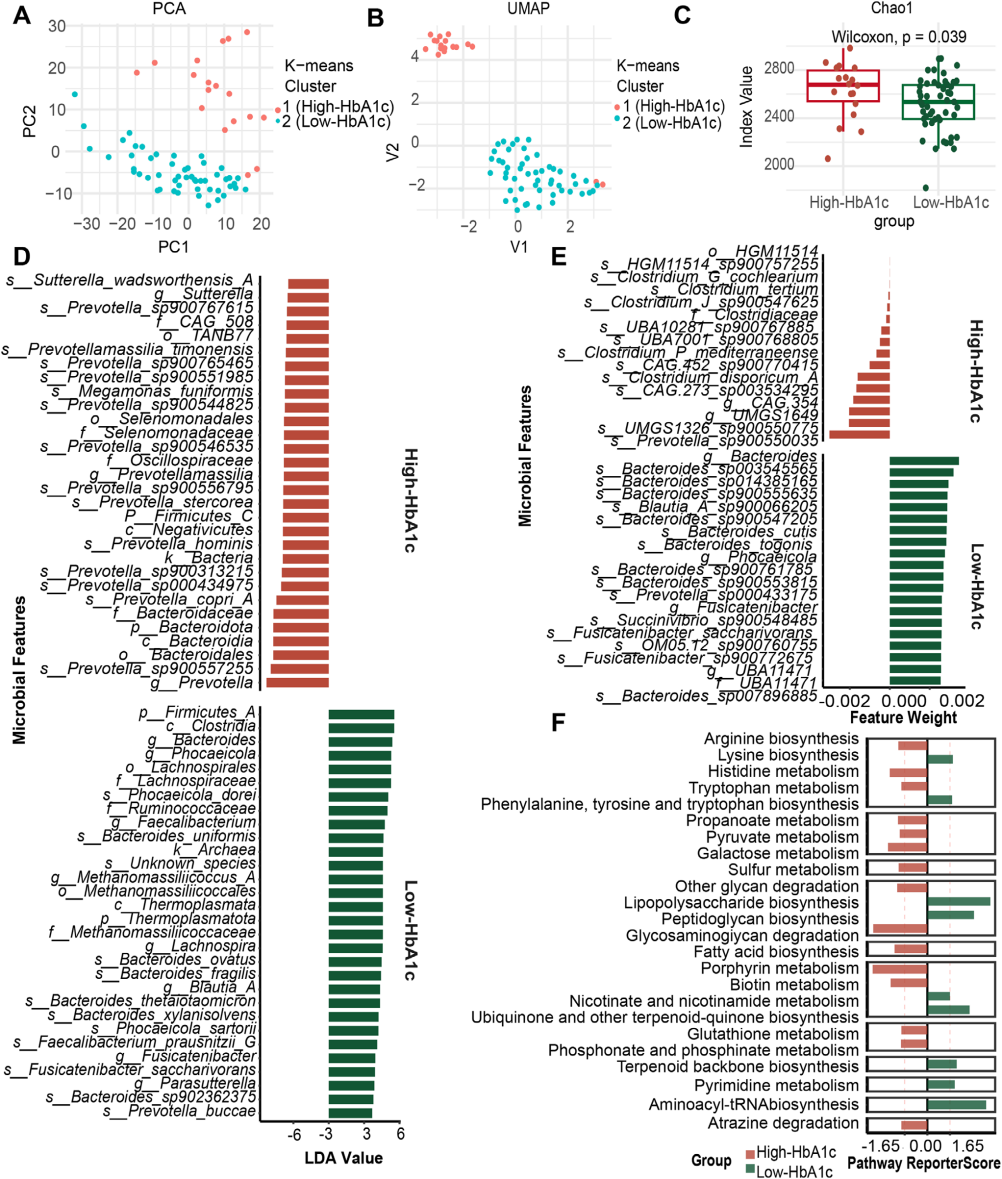
Unsupervised clustering identified High‐ and Low‐HbA1c groups based on fecal metagenomic profiles in the T1D discovery cohort. (A) Unsupervised clustering of T1D discovery cohort based on fecal metagenomic profiles using K‐means algorithm identified two distinct clusters. Dimensionality reduction with principal component analysis demonstrated clear separation between clusters. (B) Uniform manifold approximation and projection demonstrated clear separation between clusters. (C) α‐Diversity analysis using Chao1 index revealed different microbiota profile between the High‐ and Low‐HbA1c group (Wilcoxon test, *p* = 0.039). (D) Linear Discriminant Analysis Effect Size (LEfSe) analysis identified differentially enriched microbial signatures between High‐ and Low‐HbA1c groups, and top 30 signatures from each group are displayed. (E) Top discriminative microbial signatures screened by support vector machine in High‐ and Low‐HbA1c groups. (F) Enrichment pathways are based on differentially the Kyoto Encyclopedia of Genes and Genomes Orthologs of the High‐ and Low‐HbA1c groups (*n* value: the High‐HbA1c group [*n* = 18] and Low‐HbA1c group [*n* = 51]).

To investigate the heterogeneity of gut microbiota in children and adolescents with recent‐onset T1D, we used K‐means [13], an unsupervised machine‐learning algorithm renowned for its robustness, to cluster the T1D discovery cohort patients based on fecal metagenomic data. The optimal silhouette coefficient for K‐means clustering was determined to be two, with T1D patients segregated into two distinct clusters (Cluster 1 and Cluster 2). Dimensionality reduction using uniform manifold approximation and projection and principal component analysis further demonstrated clear separation between the two clusters, confirming distinct gut microbial compositions (Figure 1A,B).

Baseline clinical characteristics were then compared between the two clusters (Table 1). Cluster 1 patients showed markedly elevated HbA1c levels relative to those in Cluster 2, whereas no significant differences were detected for other clinical variables. Accordingly, Cluster 1 was designated as the High‐HbA1c group and Cluster 2 as the Low‐HbA1c group.

**TABLE 1:** Characteristics of type 1 diabetes patients identified in High‐ and Low‐HbA1c groups.

	Cluster 1 (High‐HbA1c) (*N* = 18)	Cluster 2 (Low‐HbA1c) (*N* = 51)	*p*‐value
Male	9 (50.0%)	27 (52.9%)	1.000
Age (years)	10.9 (3.96)	10.4 (3.93)	0.663
Onset age (years)	9.24 (3.55)	8.54 (3.78)	0.484
Duration (years)	1.85 [0.65; 2.55]	1.40 [0.90; 2.70]	0.575
HLA‐HR (yes)	7 (38.9%)	21 (41.2%)	1.000
HbA1c (%)	7.45 [6.85; 8.40]	6.90 [6.30; 7.60]	**0.023**
GLU (mmol/L)	8.71 [6.26; 12.0]	7.45 [5.25; 8.87]	0.062
ZnT8 (+)	10 (55.6%)	18 (35.3%)	0.220
IA2 (+)	13 (72.2%)	33 (64.7%)	0.771
GAD (+)	11 (61.1%)	35 (68.6%)	0.771
Heart rate (bpm)	87.6 (14.1)	92.7 (15.6)	0.208
SBP (mmHg)	103 (10.6)	104 (15.0)	0.859
DBP (mmHg)	66.0 [62.2; 72.5]	65.6 [59.5; 73.8]	0.848
Weight (kg)	29.8 [26.1; 49.5]	33.0 [23.8; 45.6]	0.956
Height (cm)	144 (20.6)	144 (22.7)	0.972
Waistline (cm)	57.5 [55.2; 67.9]	59.0 [53.0; 66.0]	0.652
Hipline (cm)	74.6 (12.6)	71.8 (16.2)	0.457
BMI (kg/m^2^)	15.7 [14.7;18.6]	15.8 [14.7; 18.4]	0.875
WHR	0.82 (0.06)	0.84 (0.06)	0.275
HDL‐C (mmol/L)	1.76 (0.41)	1.63 (0.43)	0.259
LDL‐C (mmol/L)	2.23 [1.88; 2.42]	2.14 [1.76; 2.72]	0.962
TG (mmol/L)	0.64 [0.54; 0.79]	0.70 [0.52; 0.96]	0.722
TC (mmol/L)	4.21 [3.79; 4.62]	4.09 [3.62; 4.75]	0.677

*Note*: Data are summarized as count (percentage), mean ± standard deviation, or median [interquartile range], as appropriate.

Abbreviations: BMI, body mass index; DBP, diastolic blood pressure (blood pressure); GAD, glutamic acid decarboxylase autoantibodies (islet autoantibodies); GLU, glucose; HbA1c, hemoglobin A1c; HDL‐C, high‐density lipoprotein cholesterol; Heart rate, beats per minute; Height, body height; Hipline, hip circumference (vital and anthropometric parameters); HLA‐HR, high‐risk human leukocyte antigen (defined by presence of two risk haplotypes: DR3, DR4, DR9, and absence of protective ones: DR8, DR11, DR12, DR15, DR16); IA2, insulinoma‐associated protein 2 autoantibodies; LDL‐C, low‐density lipoprotein cholesterol (glycemic and lipid markers); SBP, systolic blood pressure; TC, total cholesterol; TG, triglycerides; Waistline, waist circumference; Weight, body weight; WHR, waist‐to‐hip ratio (body composition); ZnT8, zinc transporter 8 autoantibodies.

### 2.2 Gut Microbial Signatures in High‐ and Low‐HbA1c Groups

We assessed α‐diversity using the Chao1 index, which revealed significantly different diversity between the two groups (Wilcoxon, *p* = 0.039; Figure 1C). A total of 115 and 125 signatures were resolved in the High‐ and Low‐HbA1c groups by Linear Discriminant Analysis Effect Size (LEfSe) analysis, respectively (Figure 1D depicts the top 30 microbial signatures from each group). After adjusting for the confounding effects of sex, age, BMI, insulin dose, and sex hormones using the Multivariate Analysis by Linear Models (MaAsLin2) model, 119 and 107 microbial signatures were identified as associated with the High‐ and Low‐HbA1c groups, respectively (false discovery rate [FDR] < 0.1). Figure S1 depicts the top 30 signatures from each group. Notably, 22 (High‐HbA1c) and 20 (Low‐HbA1c) of the top 30 signatures overlapped with those identified by LEfSe, indicating that the glycemic status‐associated gut microbial signatures remain stable even after accounting for these confounders. The top 16 and 20 microbial signatures of the support vector machine (SVM) model from the High‐ and Low‐HbA1c groups, respectively, are shown in Figure 1E. The receiver operating characteristic (ROC) value of the fivefold cross‐validation of the SVM model was 0.95.

Both LEfSe, MaAsLin2 and SVM analyses demonstrated distinct microbial signatures between the High‐ and Low‐HbA1c groups, with predominant enrichment of the phylum *Bacteroidota* and *Firmicutes*, respectively. The genera *Prevotella* (linear discriminant analysis [LDA] score = 5.59) and the phylum *Firmicutes_A* (LDA score = 4.56) were identified as the most discriminative microbiota in the High‐HbA1c and Low‐HbA1c groups, respectively. Concurrently, pathway enrichment analysis of differential KEGG Ortholog (KO) between the two groups revealed tryptophan metabolism (report score = 1.89) and phenylalanine, tyrosine, and tryptophan biosynthesis (report score = 1.81) as the significantly enriched pathways in the High‐ and Low‐HbA1c groups, respectively (Figure 1F). KEGG module‐based analysis confirmed enrichment of microbial tryptophan biosynthesis in the Low‐HbA1c group (Figure S2).

### 2.3 Serum Metabolic and Lipidomic Signatures in High‐ and Low‐HbA1c Groups

**FIGURE 2:**
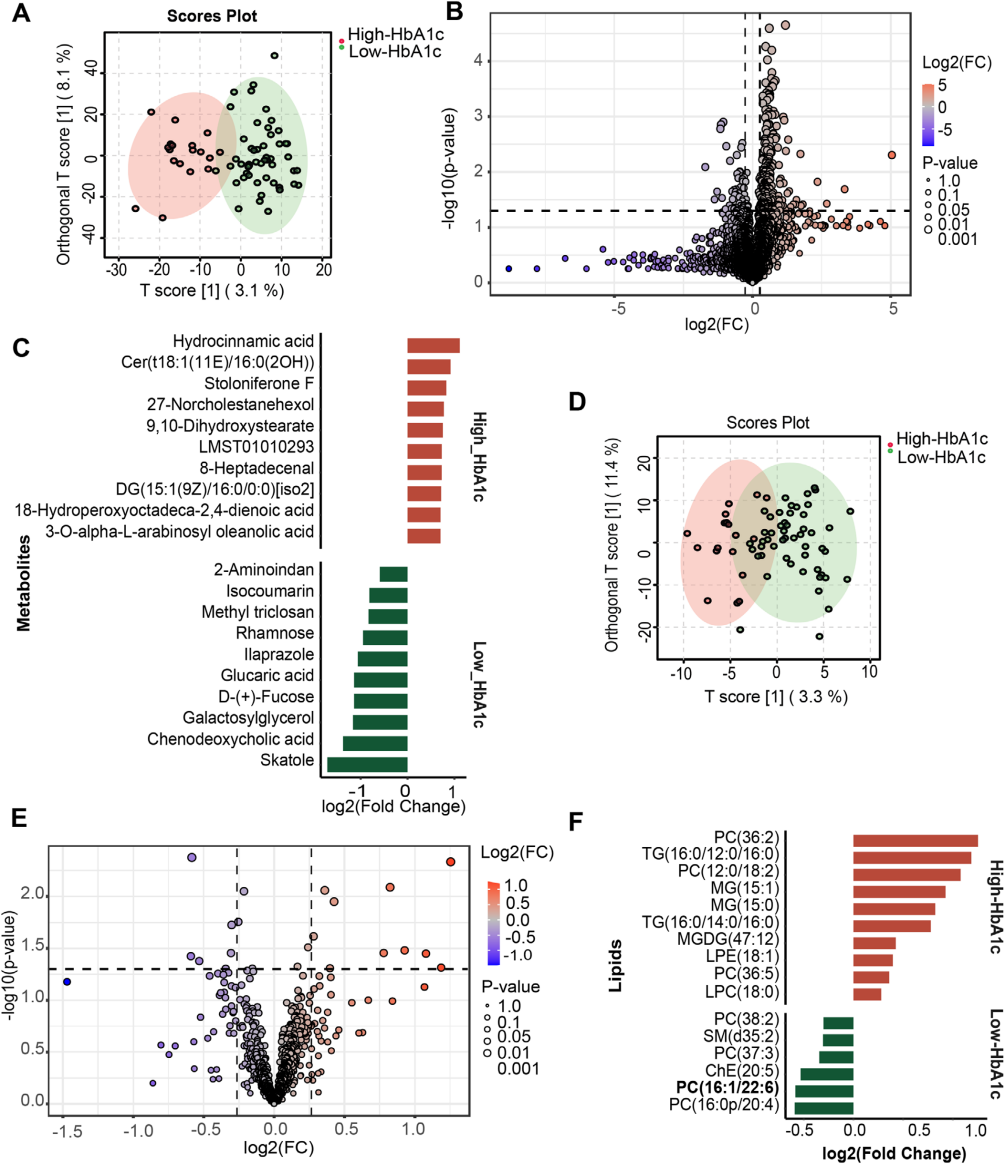
Serum metabolic and lipidomic signatures in High‐ and Low‐HbA1c Groups. (A) Orthogonal partial least squares discriminant analysis (OPLS‐DA) revealed clear separation of serum metabolomic profiles between the High‐ and Low‐HbA1c groups. (B) Volcano plots showing the serum metabolic signatures screening by fold change (>1.2) and *p* value (<0.05) using *t*‐test. (C) Top 10 metabolic signatures from the High‐ and Low‐HbA1c groups. (D) OPLS‐DA of serum lipidomic profiles revealed separation between the two groups. (E) Volcano plots showing the serum lipidomic signatures screening by fold change (>1.2) and *p* value (<0.05) using *t*‐test. (F) Top lipidomic signatures from the High‐ and Low‐HbA1c groups (*n* value: the High‐HbA1c group [*n* = 18] and Low‐HbA1c group [*n* = 51]).

A distinct separation in serum metabolomic patterns was observed between the two HbA1c groups using orthogonal projections to latent structures discriminant analysis (OPLS‐DA) (Figure 2A). Initial analysis identified 293 significantly altered metabolites (239 elevated, 54 reduced) in the High‐HbA1c group (Figure 2B). A more stringent FDR correction defined a robust set of 88 differential metabolites (73 elevated and 15 reduced). The top 10 differential metabolites are shown in Figure 2C. Compared to the High‐HbA1c group, the tryptophan metabolite skatole was the most significantly elevated metabolite in the Low‐HbA1c group. The tryptophan metabolites serotonin and L‐kynurenine were also elevated in the Low‐HbA1c groups, despite not reaching statistical significance after FDR correction (nominal *p* < 0.05; FDR *q*‐value > 0.2). Furthermore, the metabolic pathways enriched among the differential metabolic signatures between the High‐ and Low‐HbA1c groups are listed in Table S1, with a total of 12 enriched pathways identified. Notably, the most significant enrichment of metabolic signatures occurred in the tryptophan and phenylalanine metabolism pathways in the Low‐ and High‐HbA1c groups, respectively.

Similarly, lipidomic profiling by OPLS‐DA revealed distinct lipid compositions between the two groups (Figure 2D). Compared with the Low‐HbA1c group, 10 lipidomic signatures increased in the High‐HbA1c group, and six lipidomic signatures decreased (Figure 2E,F). Phosphatidylcholine (PC) (36:2) and PC (16:0p/20:4) were identified as the most significantly altered lipidomic signatures in the High‐ and Low‐HbA1c groups, respectively. Following FDR correction, the only feature to retain statistical significance was PC (16:1/22:6) (*q* = 0.09), which was elevated in the Low‐HbA1c group. None of the lipidomic features were enriched in known metabolic pathways.

### 2.4 Correlation of the Glycemic Control‐Related Gut Microbial Signatures With Serum Metabolic Signatures

To explore the potential mechanistic link between gut microbial signatures and serum metabolic signatures in glycemic control, we identified overlapping enriched pathways between differential gut microbial KOs and serum metabolic signatures (Table 2). Three pathways were found to overlap: tryptophan metabolism, porphyrin metabolism, and galactose metabolism.

**TABLE 2:** Overlapping enriched pathways of microbial and metabolic signatures between High‐ and Low‐ HbA1c groups, along with the involved KOs.

Pathway KEGG ID	Pathway name	Microbe	KOs
map00380	Tryptophan metabolism	*Alistipes communis*	K00128; K00382; K03781
map00380	Tryptophan metabolism	*Anaerostipes hadrus*	K00128; K00626
map00860	Porphyrin metabolism	*Anaerostipes hadrus*	K01698; K01845; K01885; K02492; K04720; K13542; K19221; K24866
map00380	Tryptophan metabolism	*Bacteroides cellulosilyticus*	K00382; K00658; K01667; K03781
map00380	Tryptophan metabolism	*Bacteroides nordii*	K00382; K00658; K01667; K03781
map00380	Tryptophan metabolism	*Bacteroides ovatus*	K00382; K00658; K01667; K03781
map00380	Tryptophan metabolism	*Bacteroides xylanisolvens*	K00382; K00658; K01667; K03781
map00052	Galactose metabolism	*Citrobacter portucalensis*	K00094; K00845; K00849; K00850; K00963; K00965; K01187; K01190; K01193; K01784; K01785; K01804; K01835; K02773; K02774; K02775; K07406; K08302; K12111; K12112; K16370; K16371
map00380	Tryptophan metabolism	*Enterocloster bolteae*	K00128; K00382; K00626; K01426; K01501; K03392; K07130

*Note*: The overlapping enriched pathways shared between differential gut microbial KEGG Orthologs (KOs) and serum metabolic signatures in the High‐ and Low‐HbA1c groups are shown. For each overlapping pathway (identified by KEGG ID and pathway name), the corresponding microbial species and the specific KOs involved are listed.

From the Kyoto Encyclopedia of Genes and Genomes (KEGG) annotations, 20 gut microbial signatures were identified as encoding KOs involved in the differential pathways. Of these, eight signatures contained KOs that participated in the overlapping pathways. The distribution of KOs involved in overlapping pathways between High‐ and Low‐HbA1c groups is shown in Table S2. In addition, five serum metabolic signatures associated with these shared pathways were also identified.

**FIGURE 3:**
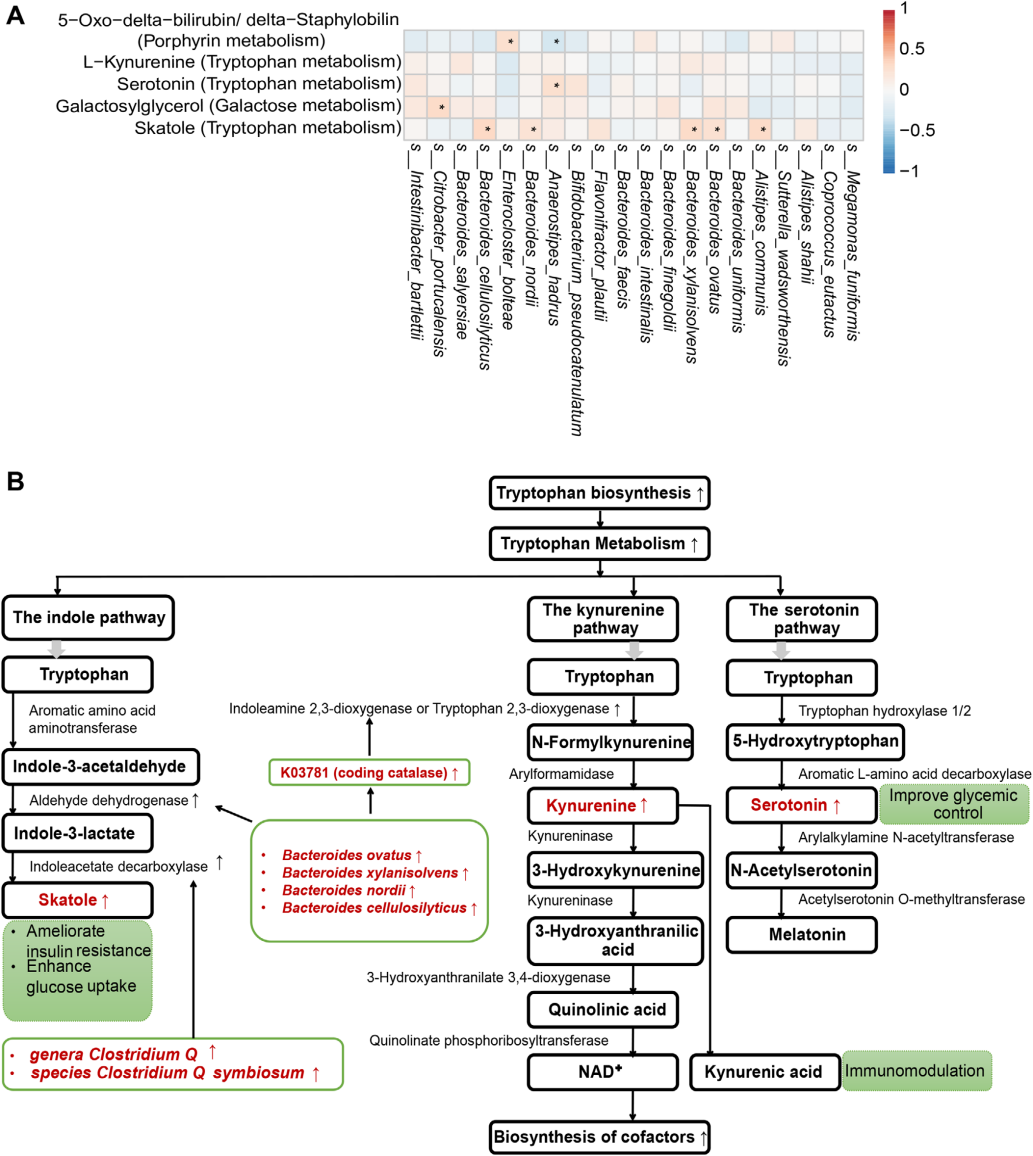
Correlation of the glycemic control‐related gut microbial signatures with serum metabolic signatures. (A) Pearson correlation analysis between gut microbial signatures and serum metabolites involved in overlapping pathways: *p ≤ 0.05. (B) The regulatory mechanisms by which the microbial and metabolic signatures are involved in the tryptophan metabolic pathway. In the figure, red highlights microbial taxa or metabolites significantly involved in tryptophan metabolism and differentially enriched between High‐ and Low‐HbA1c groups. Upward arrows indicate increased levels or enhanced activity in the Low‐HbA1c group.

Correlation analysis between the microbial and metabolic signatures participating in the overlapping pathways showed that *Bacteroides* species (*Bacteroides ovatus*, *Bacteroides xylanisolvens*, *Bacteroides nordii*, and *Bacteroides cellulosilyticus*) were positively correlated with the tryptophan metabolite skatole, with correlation coefficients of *r* = 0.26, 0.32, 0.24, and 0.35, respectively (Figure 3A). After adjusting for the potential confounding effects of sex, age, BMI, and insulin dose using multiple linear regression, *Bacteroides ovatus, Bacteroides xylanisolvens, Bacteroides nordii*, and *Bacteroides cellulosilyticus* remained positively correlated with the tryptophan metabolite skatole (*β* = 0.251, 0.290, 0.277, and 0.281, respectively), as shown in Figure S3. The possible regulatory mechanisms by which the microbial and metabolic signatures are involved in the tryptophan metabolic pathway are illustrated in Figure 3B.

### 2.5 Certain Microbial Signatures Could Distinguish Between Good and Poor Glycemic Control in the T1D Validation Cohort

**FIGURE 4:**
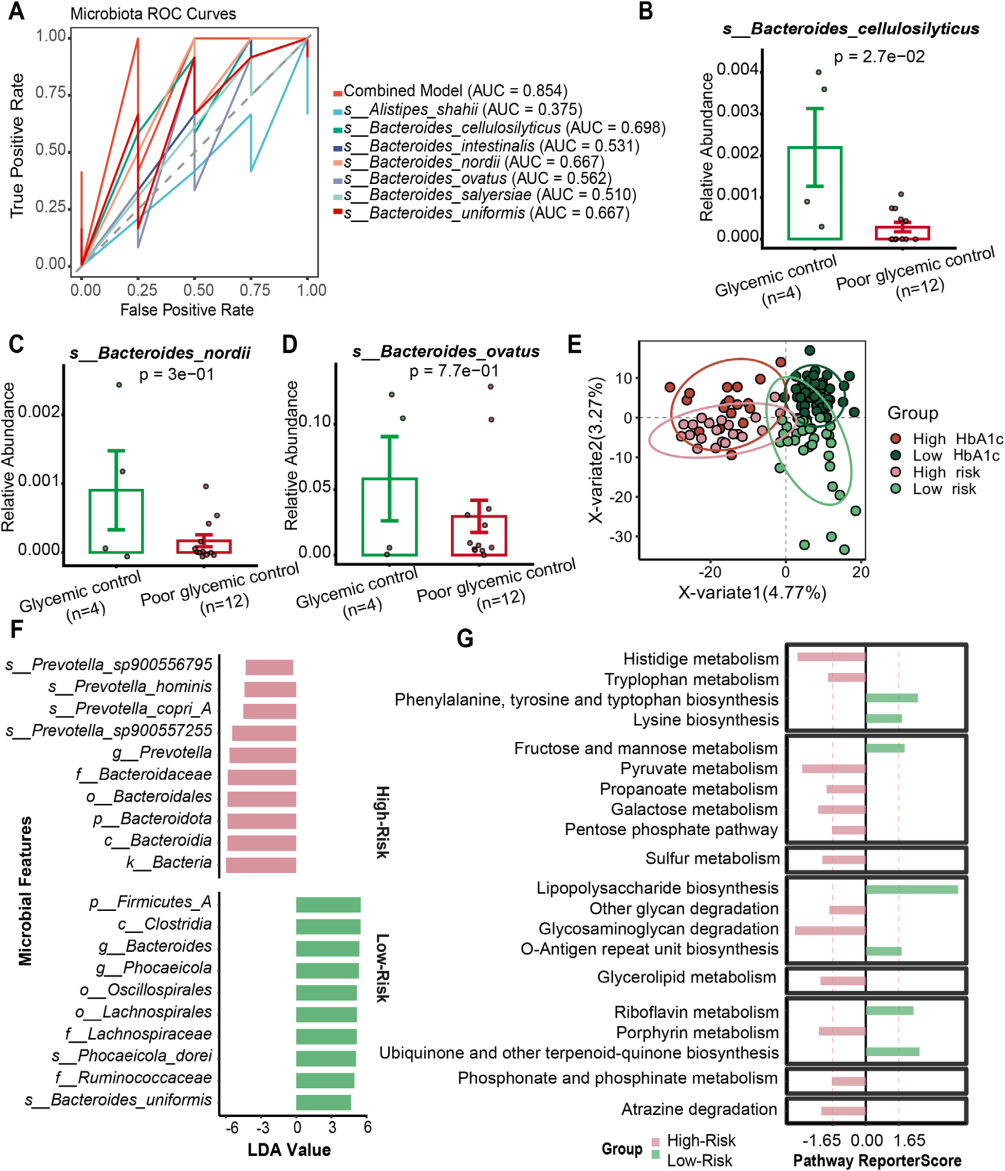
Validation of glycemic control‐related microbial signatures in the T1D validation cohort and Prediction of glycemic control‐related microbiome patterns in healthy individuals. (A) Receiver operating characteristic analysis revealed that the combined discriminatory performance of these seven microbial signatures in distinguishing the two groups achieved an AUC of 0.854, which demonstrated well discriminatory power between the glycemic control and poor glycemic control groups. (B) Relative abundance of *Bacteroides cellulosilyticus* in glycemic control and poor glycemic control groups. (C) Relative abundance of *Bacteroides nordii* in glycemic control and poor glycemic control groups. (D) Relative abundance of *Bacteroides ovatus* in glycemic control and poor glycemic control groups. (E) OPLS‐DA showing distinct gut microbiome profiles between microbiome‐defined high‐ and low‐risk groups in healthy controls. (F) LEfSe identified microbial signatures in healthy High‐ and Low‐risk groups. (G) Enriched pathways in healthy High‐ and Low‐risk groups. (*n* value: the High‐HbA1c group [*n* = 18], Low‐HbA1c group [*n* = 51], poor glycemic control group [*n* = 12], glycemic control group [*n* = 4], High‐Risk group [*n* = 22], Low‐Risk group [*n* = 34]).

The characteristics of T1D patients in glycemic control group and poor glycemic control group are shown in Table S3. We then mapped and extracted the 20 microbial signatures involved in the differential pathways from the 16S rRNA sequencing data; seven microbial signatures were detected. ROC analysis revealed that the combined discriminatory performance of these seven microbial signatures in distinguishing the two groups achieved an AUC of 0.854, which demonstrated well discriminatory power between the two different glycemic control level groups (Figure 4A). Compared with the poor glycemic control group, *Bacteroides cellulosilyticus*, *Bacteroides nordii*, and *Bacteroides ovatus* were more abundant in the glycemic control group (Figure 4B,D). Despite inherent differences in sequencing depth and primer bias between 16S rRNA and metagenomic sequencing, both platforms showed consistent patterns for the core microbiota. This concordance likely reflects our emphasis on relative between‐group differences and the high taxonomic specificity of the validated species, which together minimize cross‐platform variability. Thus, our study focuses on core differential taxa that can be robustly and reproducibly detected across sequencing modalities.

### 2.6 Prediction of Glycemic Control‐Related Microbiome Patterns in Healthy Individuals

We compared baseline characteristics between the two groups. Consistent with patterns observed in the High‐ and Low‐HbA1c groups, the high‐risk group in the healthy control group exhibited a trend toward higher HbA1c levels, though the observed difference was not statistically significant (Table S4). Subsequently, we performed gut microbiota profiling, differential signature analysis, and functional pathway analysis between the High‐ and Low‐Risk groups. OPLS‐DA revealed distinct microbial community structures between the two groups, resembling those observed between the High‐ and Low‐HbA1c groups in the T1D discovery cohort (Figure 4E). LEfSe analysis showed that 65% (50/77) and 53% (102/193) of microbial signatures in the High‐ and Low‐risk groups, respectively, overlapped with those in the High‐ and Low‐HbA1c T1D groups (Figure 4F). Similarly, 57% (4/7) and 85% (11/13) of enriched pathways overlapped between the High‐/Low‐risk groups and the High‐/Low‐HbA1c T1D groups, respectively (Figure 4G).

## 3 Discussion

### 3.1 The Phyla *Bacteroidota* and *Firmicutes* as Gut Microbial Signatures in High‐ and Low‐HbA1c Groups

*Prevotella (Bacteroidota)* and *Firmicutes_A (Firmicutes)* were identified as the most discriminative microbiota in the High‐ and Low‐HbA1c groups, respectively. Consistent with previous studies, T1D is associated with a reduced *Firmicutes*/*Bacteroidetes* ratio, characterized by increased Bacteroidetes abundance compared with healthy children [7, 14]. *Bacteroidetes* may contribute to diabetes pathogenesis by impairing epithelial barrier function, promoting inflammation, and inducing glutamic acid decarboxylase autoimmunity [15]. *Prevotella* possesses enzymatic and genetic traits for polysaccharide fermentation and may contribute to visceral hypersensitivity via enhanced carbohydrate metabolism [16]. Elevated *Prevotella* levels have been reported in T1D patients [17]. However, not all *Bacteroides* species were enriched in the High‐HbA1c group; several were more abundant in the Low‐HbA1c group, aligning with observations in well‐controlled T1D patients and healthy controls [18].

### 3.2 Serum Metabolic Signatures of Low‐HbA1c T1D Discovery Cohort Enriched in Tryptophan Metabolism

Tryptophan metabolism was the most enriched serum metabolic pathway in the Low‐HbA1c group, including skatole, L‐kynurenine, and serotonin. KEGG module‐based analysis confirmed enrichment of microbial tryptophan biosynthesis in the Low‐HbA1c group, potentially contributing to elevated serum metabolites and immunomodulatory effects. Tryptophan metabolism is critically involved in immune regulation and T1D pathogenesis [19, 20]. Additionally, its enrichment has been reported after short‐chain fatty acid‐based therapy in adult T1D patients and is associated with glycemic control [21]. It proceeds via three major routes: the microbially mediated indole pathway, the kynurenine pathway, and the serotonin pathway [22]. The indole pathway, mediated by gut microbes, produces indole derivatives that activate the aryl hydrocarbon receptor [23], inducing IL‐17 and IL‐22 to maintain gut homeostasis [24] and reduce pancreatic insulitis in NOD mice [25, 26]. The kynurenine pathway promotes regulatory T cell responses, supporting immune tolerance [27], and can be modulated by gut microbiota. Serotonin pathway activity has been linked to improved glycemic control in T1D patients treated with SSRIs29 and to enhanced insulin sensitivity [24, 28].

In this study, PC (36:2) and PC (16:0p/20:4) emerged as the most significantly altered lipids in the High‐ and Low‐HbA1c groups, respectively. Elevated diacyl‐PC (36:2) levels have been linked to reduced cardiovascular risk in T1D patients [29]. However, data on the roles of PC (36:2) and PC (16:0p/20:4) in diabetes remain limited, highlighting the need for further research.

### 3.3 Correlation Between *Bacteroides* Species and Tryptophan Metabolism

Four *Bacteroides* species (*Bacteroides ovatus*, *Bacteroides xylanisolvens*, *Bacteroides nordii*, and *Bacteroides cellulosilyticus*) were positively correlated with the tryptophan metabolite skatole, with both their abundances and serum skatole levels elevated in the Low‐HbA1c group. *Bacteroides* species can convert tryptophan into indole‐3‐lactate (IAA) [30], which may be further metabolized to skatole via indoleacetate decarboxylase [31, 32]. Although *Bacteroides* lack this enzyme, such activity has been observed in *Clostridium scatologenes* and *Lactobacillus* strains [33]. Notably, we also found increased abundance of *Clostridium Q* (LDA = 3.48, *p* < 0.05) and *Clostridium Q symbiosum* (LDA = 2.73, *p* < 0.001) in the Low‐HbA1c group, suggesting a potential synergy with *Bacteroides* in skatole production.

*Bacteroides ovatus*‐derived IAA may confer metabolic and anti‐inflammatory benefits by restoring intestinal barrier function [34] and inducing IL‐22 to protect against colitis [35]. Skatole may improve insulin sensitivity and glucose uptake by mitigating endoplasmic reticulum and oxidative stress [34]. In type 2 diabetes, higher skatole levels were linked to reduced HbA1c and fasting glucose, with statistical significance (*p* < 0.001 and *p* < 0.05, respectively), and positively correlated with *Bacteroides* abundance. Reduced skatole, linked to lower *Bacteroides* and dopa decarboxylase activity, may downregulate glucagon‐like peptide‐1 and contribute to hyperglycemia [36].

The kynurenine pathway handles about 95% of tryptophan metabolism, serving as its major metabolic route. Although L‐kynurenine (elevated in the Low‐HbA1c group) was not significantly correlated with the four *Bacteroides* species, we detected KEGG ortholog K03781 (catalase) within these species, with significantly higher expression in the Low‐HbA1c group. In the kynurenine pathway, the rate‐limiting reaction is mediated by the enzymes indoleamine 2,3‐dioxygenase (IDO) and tryptophan 2,3‐dioxygenase (TDO). Catalase activation can induce hepatic TDO expression [37], and catalase has been shown to restore IDO activity reduced by hydrogen peroxide in the ileum after *Lactobacillus johnsonii* treatment [38]. These results suggest that the four *Bacteroides* species may promote kynurenine production via catalase‐mediated enhancement of TDO and/or IDO activity, potentially supporting glycemic control in T1D. It should be noted that the proposed catalase–TDO/IDO mechanism is hypothetical and lacks support from functional‐omics or in vitro studies. Consequently, its specific role in glucose homeostasis remains to be established through targeted mechanistic experimentation.

### 3.4 *Bacteroides* Species and Predictive Microbiome Patterns for Glycemic Control

In the T1D validation cohort, seven *Bacteroides* species showed strong discriminatory power for glycemic control status, with a combined AUC of 0.854. *Bacteroides ovatus, Bacteroides nordii*, and *Bacteroides cellulosilyticus*, previously linked to tryptophan metabolism, were more abundant in the well‐controlled HbA1c group [11], suggesting their potential as therapeutic targets for glycemic regulation.

Supervised machine learning identified High‐ and Low‐Risk groups in the healthy control group based on the T1D discovery cohort clustering results. Similar microbial signatures and pathway enrichments were observed in the High‐ and Low‐HbA1c groups, suggesting gut microbiota may predict glycemic risk in healthy populations. Longitudinal studies are warranted to validate these findings.

### 3.5 Gut Microbiota as an Adjunctive Target for Glycemic Control in T1D

Insulin remains the standard therapy for children and adolescents with T1D, yet optimal glycemic control is not always achieved. Emerging evidence suggests the gut microbiota as a promising adjunctive target for glycemic control. Several cohort studies have linked *Faecalibacterium prausnitzii* [10, 12], *Prevotellaceae* spp. *SGB592* and *SGB1340* [9], *Haemophilus* [39], *Bacteroides nordii*, and *Bacteroides cellulosilyticus* [11] to lower HbA1c levels. Clinical trials with probiotics, including *Lactobacillus acidophilus* [40], *L. salivarius AP‐32, L. johnsonii MH‐68, Bifidobacterium animalis CP‐9* [41], and microbiota‐derived short‐chain fatty acids [21], have demonstrated benefits for glycemic control. Prebiotics may modulate microbiota and further improve glucose regulation by reducing gut permeability and enhancing insulin sensitivity. Despite these promising findings, mechanistic insights into the causal pathways linking gut microbiota and glycemic regulation remain limited, and existing clinical studies are often small in scale. Prebiotics represent a low‐cost, low‐risk, and potentially novel adjunctive therapy for T1D, but future large‐scale randomized controlled trials and mechanistic investigations are needed to fully elucidate their therapeutic potential.

### 3.6 Observing T1D Through an Endotype‐Based Lens

The existence of distinct T1D endotypes and their implications for precision therapy remain a subject of debate. Previous research suggests that identifying endotypes could enhance our understanding of the disease's natural history, leading to more accurate, patient‐centered treatment and follow‐up monitoring [5]. For instance, the anti‐CD20+ B‐cell monoclonal antibody rituximab has been found to be more effective in younger children at disease onset. Furthermore, children belonging to an onset‐age‐related endotype (Endotype 1) exhibit prominent pancreatic infiltration by CD8+ T cells and CD20+ B cells, highlighting the potential of endotyping for guiding precision therapy [42]. However, mechanistic links between such endotypes and specific clinical outcomes remain limited. Additionally, accurately identifying patients belonging to different T1D endotypes is still challenging. For example, one study employed an unsupervised integrative analysis of circulating immune profiles, transcriptomes, and serum metabolomic data to explore T1D endotypes, which did not identify distinct endotype patterns robustly associated with T1D disease status. However, it also did not incorporate key immune and environmental features directly linked to T1D pathogenesis, such as pancreatic characteristics and gut microbiota [43]. Given the significant potential of endotype for precision medicine, future research is needed to comprehensively characterize T1D heterogeneity and explore potential endotypes. This should leverage omics technologies and systems biology approaches to integrate multi‐dimensional data from immunology, genetics, and environmental factors [44]. Such efforts are crucial for a more holistic understanding of the mechanisms driving T1D and for paving the way toward its precise and personalized treatment.

### 3.7 Strengths and Limitations

This study is strengthened by its application of unsupervised algorithms to uncover potential heterogeneity, enabling a novel investigation of T1D endotypes from a metagenomic perspective. Additional advantages include the use of well‐characterized participants and the quantitative multi‐omics datasets. Nonetheless, several limitations of the present study should be acknowledged. First, the modest sample size may limit the power to comprehensively identify gut microbiota‐based endotypes, and thus the results currently capture only partial molecular signatures rather than the full spectrum of potential alterations. While the current cohort is sufficient to support the main conclusion, statistical power may be inadequate for subgroup analyses of certain low‐abundance species or rare taxa. Validation in larger, prospective cohorts is warranted in the future. Second, given that the gut microbiome composition fluctuates with disease stage, insulin therapy, and puberty, cross‐sectional studies alone cannot establish causality. Therefore, longitudinal cohort studies are warranted to validate the roles of glycemic control‐associated microbial signatures across the disease course, both in healthy populations and patients with T1D. Third, the cohort used for independent validation was profiled by 16S rRNA sequencing, whose lower taxonomic resolution limits our ability to validate all metagenomic findings at the precise species level. Future validation of the results from the discovery cohort will require metagenomic sequencing in larger populations. Fourth, the lack of mechanistic validation through in vitro cellular experiments or in vivo animal models precludes definitive conclusions regarding the causal relationships between *Bacteroides* species signatures, skatole production, and glycemic control in T1D. Future studies integrating larger cohorts, prospective designs, and mechanistic investigations will be essential to substantiate and extend our findings.

## 4 Conclusion

In conclusion, using a data‐driven, multi‐omics approach, we revealed heterogeneity of T1D endotypes from the gut microbiome perspective. Our findings highlight the roles of specific microbial species and metabolites in glycemic control and provide a framework for future microbiome‐targeted strategies for personalized management of T1D.

## 5 Methods

### 5.1 Study Population and Sample Collection

Participants, including T1D patients and healthy controls, were enrolled through the T1D China Registry Study in China between 2019 and 2022 (ChiCTR2000034642). This study complied with the Declaration of Helsinki. The Ethics Committee of the First Affiliated Hospital of USTC approved the study. All participants signed written informed consent. T1D patients were diagnosed by endocrinologists based on the American Diabetes Association's diagnostic criteria for T1D, while healthy controls had no history of diabetes. Exclusion criteria included gastrointestinal disorders and antibiotic or probiotic use within the past 3 months. All T1D patients received diabetes education and followed a diabetes‐specific diet [45]. In this study, participants were recruited into three cohorts, including a discovery cohort, a validation cohort, and a healthy control cohort. The T1D discovery cohort included 69 children and adolescents with recent‐onset T1D (diabetes duration within 3 years). The T1D validation cohort consisted of 16 individuals with recent‐onset T1D (diabetes duration within 5 years) used to validate the performance of the microbial signatures identified in the discovery cohort. The healthy control cohort comprised 56 individuals and was used for the preliminary investigation of the predictive potential of microbial signatures related to glycemic control. As an exploratory study, the sample size in this work is comparable to those employed in similar technical validation studies of the gut microbiota in T1D [9].

Personal and health information was collected via interviews and electronic medical records (Table 1). All participants provided fecal samples, which were transported and stored at –80°C. Metagenomic sequencing was performed on fecal samples from the T1D discovery cohort, while 16S rRNA sequencing was used for the validation cohort. Serum samples from the discovery cohort were stored at –80°C for metabolomic and lipidomic analyses.

### 5.2 Machine Learning Analysis and Statistical Analysis

Data analysis and results visualization were based on R version 4.1.1717. To address the unsupervised nature and high dimensionality of metagenomic data, K‐means clustering [13] was applied to the T1D discovery cohort to identify microbial subgroups. Prior to K‐means analyses, all metagenomic microbial relative abundance data were subjected to a centered log‐ratio transformation. This standard preprocessing for compositional data was applied to avoid the detection of mathematically driven artifacts rather than biological signals when using K‐means that assume Euclidean geometry. Then, dimensionality reduction methods, uniform manifold approximation and projection and principal component analysis, were used to visualize the enterotype heterogeneity. Unsupervised analysis clustered the T1D discovery cohort patients into two clusters with significantly different HbA1c levels (the High‐ and Low‐HbA1c group). Gut microbial, serum metabolomic, and lipidomic signatures were analyzed between the resulting High‐ and Low‐HbA1c groups (the specific procedures for multi‐omics analyses are described in detail in the corresponding Methods section).

To validate the glycemic relevance of microbial signatures, we examined gut microbiota profiles (16S rRNA sequencing) in an independent T1D validation cohort. Participants were categorized based on glycemic control: HbA1c ≥ 7% defined the poor glycemic control group, while HbA1c < 7% represented the glycemic control group. The glycemic control target of HbA1c < 7% was defined by the International Society for Pediatric and Adolescent Diabetes [46]. The diagnostic performance of microbial signatures for glycemic control was assessed in the T1D validation cohort using ROC analysis.

We further explored the distribution of glycemic control–related microbial signatures in the healthy control cohort. A SVM model, trained in the discovery cohort, was used to evaluate glycemic risk in the healthy control group. Previously, High‐ and Low‐HbA1c groups in the T1D cohort were identified via K‐means clustering, and an SVM model trained on metagenomic data achieved strong performance (AUC = 0.95). Applying this model to healthy individuals, we stratified them into high‐risk group and low‐risk group based on microbiome‐inferred glycemic regulation risk. Gut microbial profiles and signatures were analyzed across groups and subsequently compared between the high‐ and low‐HbA1c groups.

Appropriate statistical tests, including the chi‐square test, Wilcoxon rank‐sum test, and Student's *t*‐test, were applied to compare clinical variables between groups.

### 5.3 Metagenomics Next‐Generation Sequencing and Microbiota Analysis

Metagenomic sequencing of fecal samples was conducted using the MGI DNBSEQ sequencing platform with rolling circle amplification (RCA), a method that reduces sequencing errors by enabling linear amplification. After quality control, sequencing reads were assembled de novo using MEGAHIT, a computational tool designed for fast and memory‐efficient metagenomic assembly (with contigs shorter than 200 base pairs removed). MetaGeneMark, a gene prediction software for metagenomic sequences, was then applied to identify putative genes. To minimize redundancy, genes were clustered using CD‐HIT (Cluster Database at High Identity with Tolerance), a program that groups highly similar sequences. The resulting nonredundant gene catalog was functionally annotated through BLASTP (Basic Local Alignment Search Tool for Proteins) implemented via DIAMOND, an ultrafast sequence alignment software.

Subsequently, the processed data were subjected to downstream analyses by R version 4.1.1717. Alpha diversity and beta diversity metrics were calculated to evaluate differences in microbial composition. Gut microbial signatures discriminating High‐ and Low‐HbA1c groups were identified via LEfSe analysis, applying a LDA score cutoff of >2. The Benjamini–Hochberg procedure for FDR correction was applied, and all signatures demonstrated significance with a *q*‐value < 0.05. To mitigate the confounding effects of sex, age, BMI, insulin dose, and sex hormones (represented by the 16β‐hydroxyestradiol, androsterone, and 5β‐pregnane‐3α,20α‐diol, which correspond to metabolite of estrogen, progesterone, and androgen, respectively) that LEfSe cannot address, we conducted analysis using MaAsLin2. This method utilizes generalized linear models to identify phenotype‐associated microbiota while directly adjusting for specified covariates, making it robust for microbiome analysis and providing statistical validation for the signatures derived from LEfSe. We also performed a supervised learning algorithm (SVM) to screen out the microbial signatures. Functional annotation was performed against the KEGG to characterize metabolic pathways, and functional differences were further assessed by comparing KO abundances followed by pathway enrichment analysis.

### 5.4 Serum Metabolomic and Lipidomic Analysis

Untargeted liquid chromatography‐tandem mass spectrometry was employed to profile serum metabolites and lipids. Group profile analysis was visualized using OPLS‐DA through the MetaboAnalyst platform [47]. Differential features were identified by a fold change of >1.20 or <0.83 and a *p*‐value < 0.05, and were further required to have a *q*‐value < 0.2 after FDR correction. Identified metabolites were further analyzed through KEGG pathway enrichment to determine associated metabolic pathways.

### 5.5 Correlation Analysis

Initially, Pearson correlation analysis was employed to screen for associations among gut microbial, serum metabolic, and lipidomic signatures. Subsequently, to mitigate the potential confounding effects of sex, age, BMI, and insulin dose, key associations were further investigated using multiple linear regression models.

## Author Contributions

Tong Yue, Xueying Zheng, and Jianping Weng conceived the study, secured funding, and supervised the overall research process. The manuscript is originally made by Huiling Tan, who also performed the main data analyses and figure generation. Tong Yue contributed to the study design. Yu Ding, Huiling Tan, Zhaohe Gu, Xulin Wang, Jing Wang, Tian Wei, and Xiaoya Zhang contributed to clinical data collection. Lanxin Pan, Yu Shi, and Shiru Chang participated in results validation. Chuang Guo provided statistical analysis guidance throughout the project. All authors have read and approved the final manuscript.

## Funding

This work was supported by the Foundation programs supporting included Noncommunicable Chronic Diseases‐National Science and Technology Major Project (2023ZD0509100; 2023ZD0509102), the National Natural Science Foundation (82470870), and the Research Funds of Centre for Leading Medicine and Advanced Technologies of IHM (2023IHM01060), the China Postdoctoral Science Foundation (2025M782288), and the China Postdoctoral Science Foundation Anhui Joint Support Program (2025T028AH).

## Ethics Statement

Participants were enrolled through the T1D China Registry Study in China between 2019 and 2022 (ChiCTR2000034642). This study complied with the Declaration of Helsinki. The Ethics Committee of the First Affiliated Hospital of USTC approved the study (Ethical Approval No. 27 (2019) from the KY Ethics Committee). All participants signed written informed consent.

## Conflicts of Interest

The authors declare no conflicts of interest.

## Supporting information




**Supporting Table 1**: Metabolic Signatures Enriched Pathways Between High‐ and Low‐HbA1c Groups.
**Supporting Table 2**: Distribution of KOs Involved in Overlapping Enriched Pathways Across High‐ and Low‐HbA1c Groups.
**Supporting Table 3**: Characteristics of T1D patients in glycemic control group (HbA1c < 7%) and poor glycemic control group (HbA1c ≥ 7%).
**Supporting Table 4**: Characteristics of High‐ and Low‐Risk Groups in Healthy Control.
**Supporting Figure 1**: Top microbial signatures identified by Multivariate Analysis by Linear Models. Differentially abundant microbial signatures between High‐ and Low‐HbA1c groups were identified using Multivariate Analysis by Linear Models. The top 30 features from each group are displayed. Taxa highlighted in bold were also identified as significant by Linear Discriminant Analysis Effect Size analysis. (n value: the High‐HbA1c group (n = 18), Low‐HbA1c group (n = 51)).
(17×20 cm; 300 dpi).
**Supporting Figure 2**: Enrichment module analysis based on differentially KOs of the Highand Low‐HbA1c groups. The heatmap displays enriched metabolic modules derived from differential KEGG Orthologs (KOs) identified in the gut microbiome. Each row represents a specific metabolic pathway or module, and the color gradient indicates the Pathway ReporterScore (blue, enriched in High‐HbA1c group; red, enriched in Low‐HbA1c group). Only modules with significant enrichment are shown. (n value: the High‐HbA1c group (n = 18), Low‐HbA1c group (n = 51)).
(17×20 cm; 300 dpi).
**Supporting Figure 3**: Association between microbial and metabolite signatures after covariate adjustment. Correlations between microbial signatures and metabolite signatures were assessed using multiple linear regression, adjusting for sex, age, BMI, and insulin dose. Significant correlations (p < 0.05) are highlighted in red. (n value = 69).
(17×20 cm; 300 dpi).

## Data Availability

The data have been submitted to the SRA database. The accession number is PRJNA1285275 (available from October 31, 2025).
