# Supplementary material for: Microbiome‐Based Clustering Identifies Glycemic Control‐Related Subtypes in Youth With Recent‐Onset Type 1 Diabetes
Source: MedComm (2020). 2026 Mar 28;7(4):e70705. doi: 10.1002/mco2.70705 (PMC13042609; doi:10.1002/mco2.70705)
Supplement: Supplementary file 1 — Supporting Table 1: Metabolic Signatures Enriched Pathways Between High‐ and Low‐HbA1c Groups. Supporting Table 2: Distribution of KOs Involved in Overlapping Enriched Pathways Across High‐ and Low‐HbA1c Groups. Supporting Table 3: Characteristics of T1D patients in glycemic control group (HbA1c < 7%) and poor glycemic control group (HbA1c ≥ 7%). Supporting Table 4: Characteristics of High‐ and Low‐Risk Groups in Healthy Control. Supporting Figure 1: Top microbial signatures identified by Multivariate Analysis by Linear Models. Differentially abundant microbial signatures between High‐ and Low‐HbA1c groups were identified using Multivariate Analysis by Linear Models. The top 30 features from each group are displayed. Taxa highlighted in bold were also identified as significant by Linear Discriminant Analysis Effect Size analysis. (n value: the High‐HbA1c group (n = 18), Low‐HbA1c group (n = 51)). (17×20 cm; 300 dpi). Supporting Figure 2: Enrichment module analysis based on differentially KOs of the Highand Low‐HbA1c groups. The heatmap displays enriched metabolic modules derived from differential KEGG Orthologs (KOs) identified in the gut microbiome. Each row represents a specific metabolic pathway or module, and the color gradient indicates the Pathway ReporterScore (blue, enriched in High‐HbA1c group; red, enriched in Low‐HbA1c group). Only modules with significant enrichment are shown. (n value: the High‐HbA1c group (n = 18), Low‐HbA1c group (n = 51)). (17×20 cm; 300 dpi). Supporting Figure 3: Association between microbial and metabolite signatures after covariate adjustment. Correlations between microbial signatures and metabolite signatures were assessed using multiple linear regression, adjusting for sex, age, BMI, and insulin dose. Significant correlations (p < 0.05) are highlighted in red. (n value = 69). (17×20 cm; 300 dpi). [file MCO2-7-e70705-s001.pdf]

Title: Microbiome-Based Clustering Identifies Glycemic Control-Related Subtypes in Youth with Recent-Onset Type 1 Diabetes

Short running title: Glycemic Control-Related Subtypes in T1D

Huiling Tan[1] M.D., Yu Ding[1] M.D., Zhaohe Gu[1] M.D., Xulin Wang[1] M.D., Jing Wang[1] M.D., Tian Wei[1] M.D., Xiaoya Zhang[1] M.S., Lanxin Pan[1] M.D., Yu Shi[1] Ph.D., Shiru Chang[1] M.S., Chuang Guo[2] Ph.D. Jianping Weng[1,3*] M.D. Ph.D., Xueying Zheng[1*] M.D, Tong Yue[1*] Ph.D.

[1]Department of Endocrinology and Metabolism, The First Affiliated Hospital of USTC, Division of Life Sciences and Medicine, University of Science and Technology of China, Hefei, Anhui, 230001, China

[2]Department of Rheumatology and Immunology, Institute of Endocrine and Metabolic Diseases, The First Affiliated Hospital of USTC, Division of Life Sciences and Medicine, University of Science and Technology of China, Hefei, Anhui, 230001, China

[3]Department of Endocrinology and Metabolism, Centre for Leading Medicine and Advanced Technologies of IHM, The First Affiliated Hospital of USTC, Division of Life Sciences and Medicine, University of Science and Technology of China, Hefei, Anhui, 230001, China

Table S1 Metabolic Signatures Enriched Pathways Between High- and Low-HbA1c Groups

Pathway Name	Pathway KEGG ID	Metabolites	Enriched Group
alpha-Linolenic acid metabolism	map00592	Heptadecatrienal; (8Z,11Z,14Z)-8,11,14-Heptadecatrienal	High-HbA1c
Ascorbate and aldarate metabolism	map00053	Glucaric acid	Low-HbA1c
Bile secretion	map04976	Chenodeoxycholic acid	Low-HbA1c
Biosynthesis of unsaturated fatty acids	map01040	Linoleic acid<br>Nervonic acid	High-HbA1c
Fructose and mannose metabolism	map00051	Rhamnose	Low-HbA1c
Galactose metabolism	map00052	Galactosylglycerol	Low-HbA1c
Glycerolipid metabolism	map00561	Galactosylglycerol	Low-HbA1c
Linoleic acid metabolism	map00591	Linoleic acid<br>9-Oxo-ODE	High-HbA1c
Phenylalanine metabolism	map00360	Hydrocinnamic acid	High-HbA1c
Porphyrin metabolism	map00860	5-Oxo-delta-bilirubin; delta-Staphylobilin	High-HbA1c
Primary bile acid biosynthesis	map00120	Chenodeoxycholic acid	Low-HbA1c
Tryptophan metabolism	map00380	Skatole	Low-HbA1c

The 12 significantly enriched metabolic pathways identified from the differential metabolic signatures between the High- and Low-HbA1c groups are presented. For each pathway, the corresponding KEGG ID, the associated metabolites, and the group (High- or Low-HbA1c) in which the pathway was enriched are shown.

Table S2 Distribution of KOs Involved in Overlapping Enriched Pathways Across High- and Low-HbA1c Groups

KO	ALL (N=69)	High-HbA1c (N=18)	Low-HbA1c (N=51)	P-value
K00094	0.54 (0.96)	0.32 (0.37)	0.62 (1.08)	0.090
K00128	20.8 (9.16)	18.9 (9.93)	21.4 (8.88)	0.353
K00382	72.6 (29.4)	43.6 (16.5)	82.8 (26.0)	<0.001
K00626	31.9 (10.4)	26.8 (9.94)	33.6 (10.0)	0.018
K00658	16.0 (15.4)	5.40 (2.76)	19.8 (16.3)	<0.001
K00845	1.84 (2.04)	1.22 (1.19)	2.06 (2.23)	0.052
K00849	115 (16.2)	108 (14.9)	117 (16.0)	0.023
K00850	143 (22.4)	127 (15.9)	149 (21.9)	<0.001
K00963	25.0 (9.98)	22.2 (8.72)	26.0 (10.3)	0.147
K00965	47.4 (19.1)	35.6 (12.9)	51.6 (19.3)	<0.001
K01187	295 (97.6)	204 (43.9)	326 (91.3)	<0.001
K01190	675 (179)	594 (135)	703 (185)	0.011
K01193	67.9 (25.6)	53.9 (25.6)	72.8 (24.0)	0.011
K01426	4.38 (3.02)	3.53 (1.61)	4.68 (3.34)	0.061
K01501	3.66 (2.94)	4.87 (4.19)	3.23 (2.26)	0.130
K01667	20.7 (10.4)	11.9 (6.41)	23.8 (9.81)	<0.001
K01698	25.0 (11.7)	19.3 (6.33)	27.0 (12.6)	0.001
K01784	169 (30.1)	162 (26.6)	171 (31.1)	0.237
K01785	225 (53.0)	185 (31.2)	239 (52.3)	<0.001
K01835	113 (18.4)	116 (23.7)	112 (16.3)	0.581
K01845	27.4 (12.8)	24.4 (9.53)	28.4 (13.8)	0.174
K01885	97.4 (23.2)	115 (23.5)	91.3 (19.9)	0.001
K02492	18.1 (8.57)	14.0 (4.92)	19.6 (9.13)	0.002
K02773	12.0 (14.6)	13.9 (25.1)	11.3 (8.62)	0.669
K02774	17.6 (28.5)	23.0 (51.2)	15.7 (14.0)	0.563
K02775	53.3 (20.2)	65.5 (24.7)	49.0 (16.6)	0.015
K03392	0.03 (0.09)	0.00 (0.01)	0.04 (0.11)	0.016
K03781	18.8 (12.2)	10.8 (6.68)	21.7 (12.5)	<0.001
K04720	66.5 (19.6)	42.7 (14.1)	75.0 (13.3)	<0.001
K07130	24.8 (11.2)	19.0 (8.78)	26.9 (11.4)	0.004
K07406	4.13 (3.08)	2.93 (1.95)	4.56 (3.30)	0.016
K08302	16.6 (9.53)	12.3 (8.60)	18.2 (9.43)	0.020
K12111	2.58 (3.41)	3.40 (5.45)	2.29 (2.33)	0.411
K12112	2.53 (3.16)	1.95 (2.16)	2.73 (3.44)	0.272
K13542	17.5 (8.69)	14.0 (6.08)	18.8 (9.17)	0.018
K16370	1.76 (1.83)	1.38 (1.22)	1.89 (2.00)	0.212
K16371	6.15 (4.36)	4.46 (2.62)	6.75 (4.71)	0.014
K19221	65.5 (24.4)	58.9 (23.1)	67.9 (24.7)	0.172
K24866	23.5 (10.7)	19.8 (9.57)	24.7 (10.9)	0.076
K01804	60.7 (13.7)	60.8 (15.0)	60.6 (13.3)	0.976

The abundance distribution of KEGG Orthologs (KOs) identified in the overlapping enriched pathways is shown for all participants (N=69) and stratified by the High-HbA1c (N=18) and Low-HbA1c (N=51) groups. Data are presented as mean (standard deviation). P-values indicate the significance of differences between the High- and Low-HbA1c groups.

Table S3 Characteristics of T1D patients in glycemic control group (HbA1c < 7%) and poor glycemic control group (HbA1c ⩾ 7%)

	Poor glycemic control group (N=12)	Glycemic control group (N=4)	P-value
HLA-HR (yes)	7 (58.3%)	1 (25.0%)	0.569
Male	6 (50.0%)	1 (25.0%)	0.585
Age (years)	12.0 [9.75;15.8]	18.5 [10.8;27.0]	0.360
Age of onset (years)	8.00 [5.75;13.8]	16.0 [7.75;24.8]	0.273
Diabetes duration (years)	3.70 (1.10)	3.03 (0.99)	0.300
BMI (kg/m2)	19.2 (3.33)	18.4 (2.47)	0.653
HbA1c (%)	9.24 (2.12)	5.82 (0.21)	**<0.001**
Fasting C-peptide (nmol/l)	0.02 [0.02;0.02]	0.08 [0.02;0.18]	0.111
IA (+)	7 (58.3%)	2 (50.0%)	1.000
GAD (+)	4 (33.3%)	2 (50.0%)	0.604
ZnT8 (+)	3 (25.0%)	1 (25.0%)	1.000
IA2 (+)	3 (25.0%)	1 (25.0%)	1.000
TC (mmol/l)	4.39 [3.87;4.71]	4.76 [4.20;5.27]	0.504
TG (mmol/l)	0.66 (0.18)	0.74 (0.14)	0.439
HDL-C (mmol/l)	1.51 (0.31)	1.67 (0.15)	0.216
LDL-C (mmol/l)	2.36 [2.25;2.90]	2.54 [2.20;2.86]	0.671

Data are presented as number (%), mean (standard deviation), and median [interquartile range]. Abbreviations: HLA-HR: human leukocyte antigen-high risk (Patients with two risk haplotypes (DR3, DR4, DR9 haplotype) and without protective haplotypes (DR8, DR11, DR12, DR15, DR16 haplotype) were thus categorized as “high risk”); HbA1c: haemoglobin A1c; GLU: glucose; ZnT8: zinc transporter 8 autoantibodies; IA2: insulinoma-associated protein 2 autoantibodies; GAD: glutamic acid decarboxylase autoantibodies; BMI: body mass index; HDL-C: high-density lipoprotein cholesterol; LDL-C: low-density lipoprotein cholesterol; TG: triglycerides; TC: total cholesterol.

Table S4 Characteristics of High- and Low-Risk Groups in Healthy Control

	High-Risk (N=22)	Low-Risk (N=34)	P-value
Male	10 (45.5%)	19 (55.9%)	0.625
Age (years)	11.9 (4.86)	9.98 (4.25)	0.146
HLA-HR (yes)	0 (0.00%)	2 (5.88%)	0.514
HbA1c (%)	5.45 [5.40;5.60]	5.40 [5.30;5.50]	0.228
GLU (mmol/l)	4.79 (0.46)	4.73 (0.51)	0.630
Heart rate (bpm)	88.5 [76.2;92.4]	88.0 [77.7;93.8]	0.718
SBP (mmHg)	114 [106;124]	110 [102;124]	0.718
DBP (mmHg)	69.5 (7.57)	69.4 (7.71)	0.959
Weight (kg)	41.0 (12.6)	37.0 (16.7)	0.311
Height (cm)	152 (21.0)	142 (24.0)	0.124
Waistline (cm)	64.4 (6.80)	61.9 (10.6)	0.292
Hipline (cm)	80.0 (9.55)	75.4 (13.0)	0.128
BMI (kg/m2)	17.3 [16.1;17.9]	16.7 [15.4;18.0]	0.374
WHR	0.81 (0.06)	0.82 (0.04)	0.288
HDL-C (mmol/l)	1.58 (0.17)	1.67 (0.21)	0.082
LDL-C (mmol/l)	1.97 [1.80;2.19]	2.03 [1.86;2.29]	0.369
TG (mmol/l)	0.62 [0.54;0.67]	0.66 [0.59;0.72]	0.347
TC (mmol/l)	3.91 [3.75;4.04]	4.00 [3.85;4.51]	0.214

Data are presented as number (%), mean (standard deviation), and median [interquartile range]. Abbreviations: HLA-HR: human leukocyte antigen-high risk (Patients with two risk haplotypes (DR3, DR4, DR9 haplotype) and without protective haplotypes (DR8, DR11, DR12, DR15, DR16 haplotype) were thus categorized as “high risk”); HbA1c: haemoglobin A1c; GLU: glucose; Heart rate: heart rate; SBP: systolic blood pressure; DBP: diastolic blood pressure; Weight: body weight; Height: body height; Waistline: waist circumference; Hipline: hip circumference; BMI: body mass index; WHR: waist-to-hip ratio; HDL-C: high-density lipoprotein cholesterol; LDL-C: low-density lipoprotein cholesterol; TG: triglycerides; TC: total cholesterol.

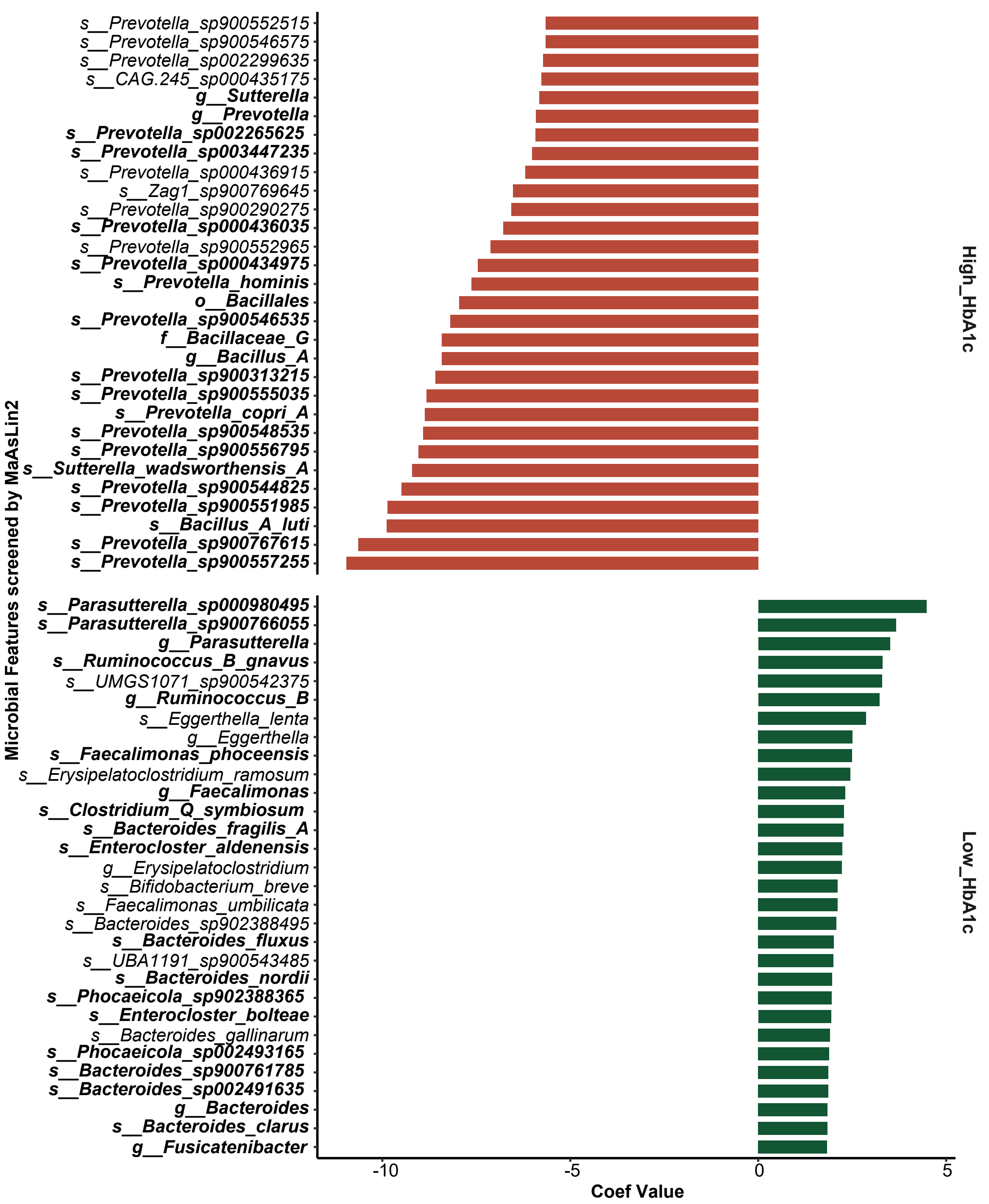

s__Prevotella_sp900552515
s__Prevotella_sp900546575
s__Prevotella_sp002299635
s__CAG.245_sp000435175
g__Sutterella
g__Prevotella
s__Prevotella_sp002265625
s__Prevotella_sp003447235
s__Prevotella_sp000436915
s__Zag1_sp900769645
s__Prevotella_sp900290275
s__Prevotella_sp000436035
s__Prevotella_sp900552965
s__Prevotella_sp000434975
s__Prevotella_hominis
o__Bacillales
s__Prevotella_sp900546535
f__Bacillaceae_G
g__Bacillus_A
s__Prevotella_sp900313215
s__Prevotella_sp900555035
s__Prevotella_copri_A
s__Prevotella_sp900548535
s__Prevotella_sp900556795
s__Sutterella_wadsworthensis_A
s__Prevotella_sp900544825
s__Prevotella_sp900551985
s__Bacillus_A_luti
s__Prevotella_sp900767615
s__Prevotella_sp900557255
High_HbA1c
s__Parasutterella_sp000980495
s__Parasutterella_sp900766055
g__Parasutterella
s__Ruminococcus_B_gnavus
s__UMGS1071_sp900542375
g__Ruminococcus_B
s__Eggerthella_lenta
g__Eggerthella
s__Faecalimonas_phoceensis
s__Erysipelatoclostridium_ramosum
g__Faecalimonas
s__Clostridium_Q_symbiosum
s__Bacteroides_fragilis_A
s__Enterocloster_aldenensis
g__Erysipelatoclostridium
s__Bifidobacterium_breve
s__Faecalimonas_umbilicata
s__Bacteroides_sp902388495
s__Bacteroides_fluxus
s__UBA1191_sp900543485
s__Bacteroides_nordii
s__Phocaeicola_sp902388365
s__Enterocloster_bolteae
s__Bacteroides_gallinarum
s__Phocaeicola_sp002493165
s__Bacteroides_sp900761785
s__Bacteroides_sp002491635
g__Bacteroides
s__Bacteroides_clarus
g__Fusicatenibacter
Low_HbA1c
Microbial Features screened by MaAsLin2
-10
-5
0
5
Coef Value

Figure S1. Top microbial signatures identified by Multivariate Analysis by Linear Models. Differentially abundant microbial signatures between High- and Low-HbA1c groups were identified using Multivariate Analysis by Linear Models. The top 30 features from each group are displayed. Taxa highlighted in bold were also identified as significant by Linear Discriminant Analysis Effect Size analysis. (n value: the High-HbA1c group (n=18), Low-HbA1c group (n=51))

(17×20 cm; 300 dpi)

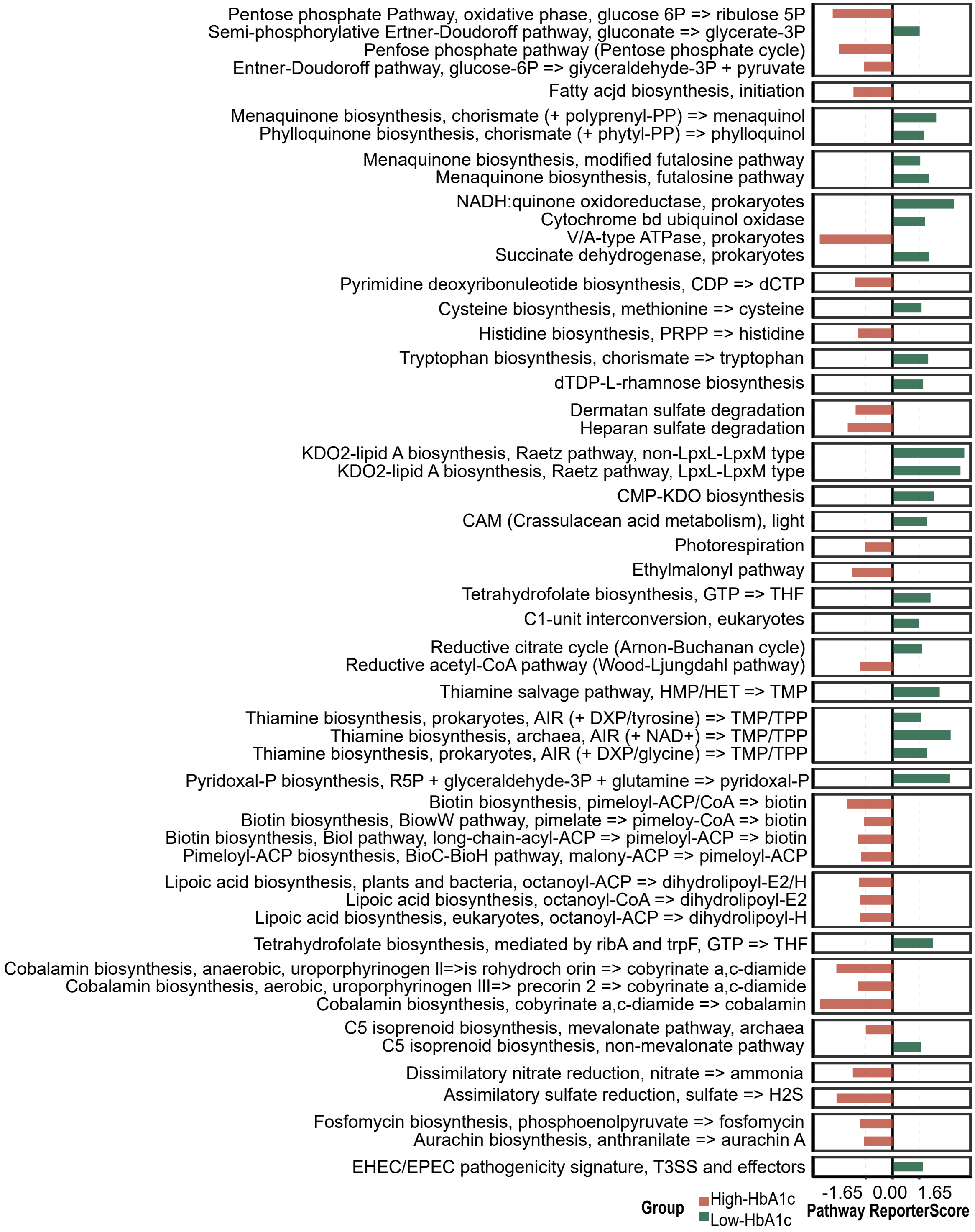

Pentose phosphate Pathway, oxidative phase, glucose 6P => ribulose 5P
Semi-phosphorylative Ertner-Doudoroff pathway, gluconate => glycerate-3P
Penfose phosphate pathway (Pentose phosphate cycle)
Entner-Doudoroff pathway, glucose-6P => giyceraldehyde-3P + pyruvate
Fatty acjd biosynthesis, initiation
Menaquinone biosynthesis, chorismate (+ polyprenyl-PP) => menaquinol
Phylloquinone biosynthesis, chorismate (+ phytyl-PP) => phylloquinol
Menaquinone biosynthesis, modified futalosine pathway
Menaquinone biosynthesis, futalosine pathway
NADH:quinone oxidoreductase, prokaryotes
Cytochrome bd ubiquinol oxidase
V/A-type ATPase, prokaryotes
Succinate dehydrogenase, prokaryotes
Pyrimidine deoxyribonuleotide biosynthesis, CDP => dCTP
Cysteine biosynthesis, methionine => cysteine
Histidine biosynthesis, PRPP => histidine
Tryptophan biosynthesis, chorismate => tryptophan
dTDP-L-rhamnose biosynthesis
Dermatan sulfate degradation
Heparan sulfate degradation
KDO2-lipid A biosynthesis, Raetz pathway, non-LpxL-LpxM type
KDO2-lipid A biosynthesis, Raetz pathway, LpxL-LpxM type
CMP-KDO biosynthesis
CAM (Crassulacean acid metabolism), light
Photorespiration
Ethylmalonyl pathway
Tetrahydrofolate biosynthesis, GTP => THF
C1-unit interconversion, eukaryotes
Reductive citrate cycle (Arnon-Buchanan cycle)
Reductive acetyl-CoA pathway (Wood-Ljungdahl pathway)
Thiamine salvage pathway, HMP/HET => TMP
Thiamine biosynthesis, prokaryotes, AIR (+ DXP/tyrosine) => TMP/TPP
Thiamine biosynthesis, archaea, AIR (+ NAD+) => TMP/TPP
Thiamine biosynthesis, prokaryotes, AIR (+ DXP/glycine) => TMP/TPP
Pyridoxal-P biosynthesis, R5P + glyceraldehyde-3P + glutamine => pyridoxal-P
Biotin biosynthesis, pimeloyl-ACP/CoA => biotin
Biotin biosynthesis, BiowW pathway, pimelate => pimeloy-CoA => biotin
Biotin biosynthesis, Biol pathway, long-chain-acyl-ACP => pimeloyl-ACP => biotin
Pimeloyl-ACP biosynthesis, BioC-BioH pathway, malony-ACP => pimeloyl-ACP
Lipoic acid biosynthesis, plants and bacteria, octanoyl-ACP => dihydrolipoyl-E2/H
Lipoic acid biosynthesis, octanoyl-CoA => dihydrolipoyl-E2
Lipoic acid biosynthesis, eukaryotes, octanoyl-ACP => dihydrolipoyl-H
Tetrahydrofolate biosynthesis, mediated by ribA and trpF, GTP => THF
Cobalamin biosynthesis, anaerobic, uroporphyrinogen ll=>is rohydroch orin => cobyrinate a,c-diamide
Cobalamin biosynthesis, aerobic, uroporphyrinogen III=> precorin 2 => cobyrinate a,c-diamide
Cobalamin biosynthesis, cobyrinate a,c-diamide => cobalamin
C5 isoprenoid biosynthesis, mevalonate pathway, archaea
C5 isoprenoid biosynthesis, non-mevalonate pathway
Dissimilatory nitrate reduction, nitrate => ammonia
Assimilatory sulfate reduction, sulfate => H2S
Fosfomycin biosynthesis, phosphoenolpyruvate => fosfomycin
Aurachin biosynthesis, anthranilate => aurachin A
EHEC/EPEC pathogenicity signature, T3SS and effectors
-1.65 0.00 1.65
Pathway ReporterScore
Group
High-HbA1c
Low-HbA1c

Figure S2. Enrichment module analysis based on differentially KOs of the High- and Low-HbA1c groups. The heatmap displays enriched metabolic modules derived from differential KEGG Orthologs (KOs) identified in the gut microbiome. Each row represents a specific metabolic pathway or module, and the color gradient indicates the Pathway ReporterScore (blue, enriched in High-HbA1c group; red, enriched in Low-HbA1c group). Only modules with significant enrichment are shown. (n value: the High-HbA1c group (n=18), Low-HbA1c group (n=51))

(17×20 cm; 300 dpi)

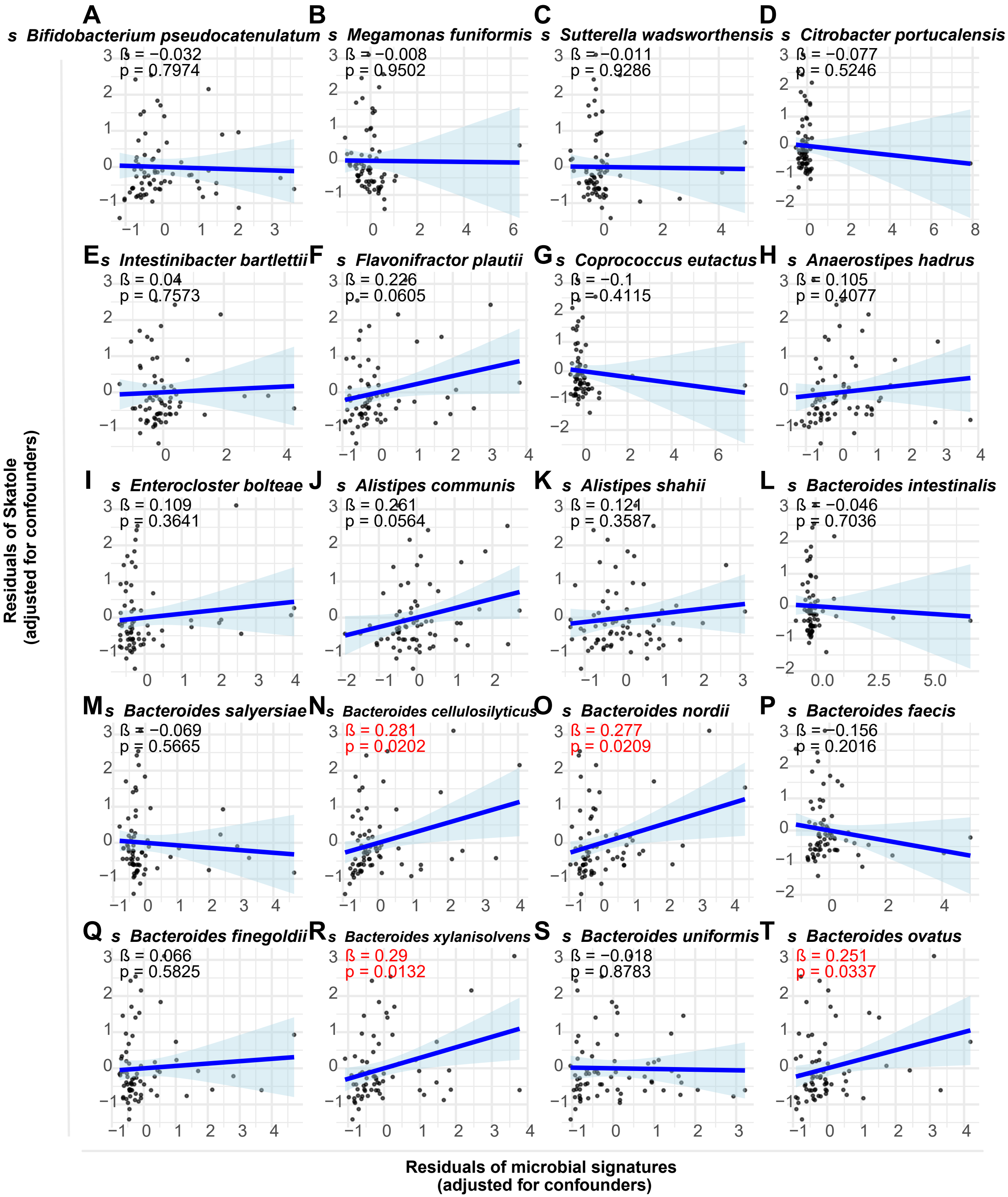
A
s Bifidobacterium pseudocatenulatum
ß = −0.032
p = 0.7974
B
s Megamonas funiformis
ß = −0.008
p = 0.9502
C
s Sutterella wadsworthensis
ß = −0.011
p = 0.9286
D
s Citrobacter portucalensis
ß = −0.077
p = 0.5246
E
s Intestinibacter bartlettii
ß = 0.04
p = 0.7573
F
s Flavonifractor plautii
ß = 0.226
p = 0.0605
G
s Coprococcus eutactus
ß = −0.1
p = 0.4115
H
s Anaerostipes hadrus
ß = 0.105
p = 0.4077
I
s Enterocloster bolteae
ß = 0.109
p = 0.3641
J
s Alistipes communis
ß = 0.261
p = 0.0564
K
s Alistipes shahii
ß = 0.121
p = 0.3587
L
s Bacteroides intestinalis
ß = −0.046
p = 0.7036
M
s Bacteroides salyersiae
ß = −0.069
p = 0.5665
N
s Bacteroides cellulosilyticus
ß = 0.281
p = 0.0202
O
s Bacteroides nordii
ß = 0.277
p = 0.0209
P
s Bacteroides faecis
ß = −0.156
p = 0.2016
Q
s Bacteroides finegoldii
ß = 0.066
p = 0.5825
R
s Bacteroides xylanisolvens
ß = 0.29
p = 0.0132
S
s Bacteroides uniformis
ß = −0.018
p = 0.8783
T
s Bacteroides ovatus
ß = 0.251
p = 0.0337
Residuals of Skatole
(adjusted for confounders)
Residuals of microbial signatures
(adjusted for confounders)

Figure S3. Association between microbial and metabolite signatures after covariate adjustment. Correlations between microbial signatures and metabolite signatures were assessed using multiple linear regression, adjusting for sex, age, BMI, and insulin dose. Significant correlations ($p < 0.05$) are highlighted in red. (n value=69)

(17×20 cm; 300 dpi)
